# Extracts Characterization and In Vitro Evaluation of Potential Immunomodulatory Activities of the Moss *Hypnum cupressiforme* Hedw.

**DOI:** 10.3390/molecules25153343

**Published:** 2020-07-23

**Authors:** Tanja M. Lunić, Mariana M. Oalđe, Marija R. Mandić, Aneta D. Sabovljević, Marko S. Sabovljević, Uroš M. Gašić, Sonja N. Duletić-Laušević, Bojan Dj. Božić, Biljana Dj. Božić Nedeljković

**Affiliations:** 1 Institute of Physiology and Biochemistry “Ivan Djaja”, Faculty of Biology, University of Belgrade, 11000 Belgrade, Serbia; b3018_2019@stud.bio.bg.ac.rs (T.M.L.); b3022_2019@stud.bio.bg.ac.rs (M.R.M.); 2 Institute of Botany and Botanical Garden “Jevremovac”, Faculty of Biology, University of Belgrade, 11000 Belgrade, Serbia; marianao@bio.bg.ac.rs (M.M.O.); aneta@bio.bg.ac.rs (A.D.S.); marko@bio.bg.ac.rs (M.S.S.); sduletic@bio.bg.ac.rs (S.N.D.-L.); 3 Department of Plant Physiology, Institute for Biological Research “Sinisa Stankovic”, National Institute of Republic of Serbia, University of Belgrade, Bulevar Despota Stefana 142, 11060 Belgrade, Serbia; uros.gasic@ibiss.bg.ac.rs

**Keywords:** moss extract, *Hypnum cupressiforme*, antioxidant, anti-neuroinflammatory/antineurodegenerative, antidiabetic, antitumor activity

## Abstract

Recently, there has been an increasing interest in the chemistry and biological potential of mosses, since a large number of biologically active compounds have been found within these species. This study aimed at examining the chemical composition and immunomodulatory potential (antioxidant, antidiabetic, anti-neuroinflammatory/antineurodegenerative, and antitumor activities) of moss *Hypnum cupressiforme* Hedw. extracts. Corresponding extracts have been obtained applying Soxhlet extractor. The chemical characterization was performed using spectrophotometric assays and liquid chromatography–mass spectrometry (LC-MS). The extracts were analyzed for antioxidant activity and for inhibitory activities on α-glucosidase, α-amylase, acetylcholinesterase, and tyrosinase. Additionally, extracts were tested against four cell lines—MRC-5, BV2, HCT-116, and MDA-MB-231—for antitumor and anti-inflammatory activities. Chemical analysis of extracts revealed the presence of flavonoids, phenolic acids, and triterpenoids. Major compounds identified by LC-MS in *H. cupressiforme* were kaempferol and five phenolic acids: *p*-hydroxybenzoic, protocatechuic, *p*-coumaric, gallic, and caffeic acid. According to biochemical assays the investigated extracts exhibited significant immunomodulatory potential. Significant antiproliferative potential against MDA-MB-231 cells has been observed together with the promising anti-neuroinflammatory application. The obtained data suggest that moss *H. cupressiforme* is a valuable natural source of biologically active compounds with potential application in the pharmaceutical industry.

## 1. Introduction

Bryophytes belong to the second largest group of plants with an estimated 23,000 species worldwide [1]. They are generally classified into three phyla: Bryophyta (mosses), Marchantiophyta (liverworts), and Anthocerotophyta (hornworts). The phylum Bryophyta consists of six classes among which the most abundant is Bryopsida [2]. One of the moss species belonging to this class is *Hypnum cupressiforme* Hedw. (Hypnaceae, Hypnales), commonly known as the cypress-leaved plait-moss or simply hypnum moss. This moss can be found within almost all continents and terrestrial habitat types. It grows on versatile surfaces like logs, walls, or rocks. This moss is typically 2–10 cm long and pleurocarpous, with branched stems that are forming dense green mats. *H. cupressiforme* is a highly polymorphic species with a great number of varieties [3].

The representatives of bryophytes are commonly used in traditional medicine all over the world. A moss belonging to the Hypnaceae family, *Taxiphyllum taxirameum*, has been used in traditional Chinese medicine for the treatment of various inflammation-related conditions, ulcers, skin injuries, and for the treatment of bleeding [4]. Species from the genus *Hypnum* were used for stuffing pillows and mattresses [4], which can be related to effects such as influence on sleeping quality and may indicate the antimicrobial effect. There is evidence that *H. cupressiforme* expresses good antimicrobial, antioxidant, and antiproliferative activities [5,6]. Additionally, *H. cupressiforme* was used in different parts of the world for decoration purposes and costume making [4,7].

The chemistry of bryophytes has been overlooked earlier due to their small dimensions, amount of available material, and difficulties with identification [8]. However, recently, with the development of analytical techniques, the interest in bryophyte chemistry has increased [9]. Studies about bryophyte chemical composition are usually focused on their secondary metabolites, which can be divided into two main groups: polyphenols and lipids [9]. The majority of secondary metabolites from mosses belong to flavonoids, terpenoids, and bibenzyls. In addition, there are also compounds such as fatty acids and acetophenols [10]. It is crucial to emphasize that the quantitative and even qualitative composition of the secondary metabolites may be influenced by the environment. It is therefore important to note that moss used in this study was collected from the Vršačke Planine Mts., that is an independent geomorphological unit in southeastern Banat representing a remnant block of the old Pannonian massif [11] with specific microenvironmental factors (the amount of incident light, the degree of moisture, and the range of temperatures). That is of additional specificity and significance in the study of chemical and biological properties of mosses from this area.

The pharmaceutical industry is constantly developing novel pharmacologically active compounds. Medicinal plants are widely used as alternative therapeutic tools for the prevention or adjuvant therapy of various diseases. Among many other plants, bryophytes are recognized as promising sources of new biologically active compounds [8,12,13]. Some of the bryophytes secondary metabolites exhibit diverse biological activities such as cytotoxicity, antimicrobial, antifungal, antitumor, antioxidant, anti-inflammatory, and many others [1,6]. The biological activity of a certain plant extract depends on several factors such as chemical composition and structural configuration of functional groups, but also on potential synergistic or antagonistic interactions between compounds within the extract [14]. 

The aim of this study was to examine the chemical composition, antioxidant, antidiabetic, anti-neuroinflammatory/antineurodegenerative, and antitumor potential of the moss *H. cupressiforme* extracts in vitro. Previous studies about the biological potential of *H. cupressiforme* are infrequent, reporting good antimicrobial, antioxidant, and antiproliferative activities of this species [5,6]. Additionally, this is a complex and rather genetically variable species. Therefore, this investigation, to the best of our knowledge, is the first regarding the evaluation of potential biological activities of typical moss *H. cupressiforme* originated from the unique island low-mountain area, namely, the Vršačke Planine Mts., Serbia. 

## 2. Results

### 2.1. Extraction and Chemical Characterization

In order to determine appropriate extraction time, the optimization of extraction was performed. During extraction in Soxhlet apparatus, the absorbance of the ethanolic extract was monitored every 2 h by UV spectrophotometry (UV–Vis 1700, Shimadzu, Kyoto, Japan). The maximum absorption point was reached after 10 h of extraction. This time was set as the optimum time and was used for all other solvents. Table 1 presents the solvents used for the extraction process and weights of the dry moss and crude extracts (further extract). The percentage of extraction yield ranged from 0.6% for ethyl acetate extract to 26.3% for the aqueous one. The aqueous extract was found to have a higher yield in comparison with the other extracts.

The results of Total Phenolic Content (TPC), Total Phenolic Acid Content (TPAC), Total Flavonoid Content (TFC), Total Flavonol Content (TFlC), and Total Triterpenoid Content (TTC) are presented in Table 2.

Among the tested extracts, E3 and E4 exhibited the highest TPC values—15.3 and 18.2 mg gallic acid equivalents (GAE) per g of extract. The results of the TPAC analysis indicated that the highest concentration of phenolic acids was present in E3—339.9 mg caffeic acid equivalents (CAE) per g of extract. Total Flavonoid Content (TFC) analysis of *H. cupressiforme* extracts showed the greatest flavonoid content in E3—58.9 mg quercetin equivalents (QE) per g of extract. Regarding TFlC, it was found that only ethyl acetate extract has a considerable amount of flavonols—14.1 mg QE/g extract. Extract E3 was found to possess the highest amount of TTC—236.0 mg ursolic acid equivalents (UAE) per g of extract. The other three extracts had lower TTC. The results from the determination of total coumarin content indicate that there is no considerable amount of these compounds in any of the investigated extracts (data not shown). 

#### LC-MS Analysis

To identify the specific chemical profile of all examined moss extracts (E1—ethanolic extract, E2—aqueous-ethanolic extract, E3—ethyl acetate extract, and E4—aqueous extract.), LC-MS chromatography was applied. The analysis confirmed the presence of 14 compounds (Table 3), including six phenolic acids: gallic acid, protocatechuic acid, 5-*O*-caffeoylquinic acid, *p*-hydroxybenzoic acid, caffeic acid, *p*-coumaric acid, and eight flavonoids: quercetin 3-*O*-rutinoside, quercetin 3-*O*-glucoside, isorhamnetin 3-*O*-glucoside, eriodictyol, apigenin, naringenin, kaempferol, and acacetin.

The predominant compounds of E1 extract were kaempferol, *p*-hydroxybenzoic acid, protocatechuic acid, and *p*-coumaric acid. Phytochemical characteristics of E2 extract proved to be very similar to the ones of the E1 extract. However, concentrations of the compounds in the E2 extract were slightly lower than in the E1 extract. On the contrary, E3 and E4 extracts demonstrated different phenolic profiles, compared to E1 and E2 extracts, but also compared to each other. Compounds with the highest concentrations in E3 extract were *p*-hydroxybenzoic acid and protocatechuic acid, while gallic acid and *p*-coumaric acid were found in lower concentrations. Extract E4 contained high concentrations of *p*-hydroxybenzoic and *p*-coumaric acids, followed by protocatechuic, gallic, and caffeic acids.

### 2.2. Biochemical Analysis of Moss Extracts

#### 2.2.1. Evaluation of Antioxidant Activity

The radical scavenging activity of moss extracts, measured with 2,2-diphenyl-1-picrylhydrazyl (DPPH) assay, was lower than the activity of all standard substances. According to the results, E3 and E4 extracts at the concentration of 1000 µg/mL had moderate scavenging activity (23.8 and 19.2%, respectively) compared to standard substances 3,5-di-tert-butyl-4-hydroxytoluene (BHT), 2-tert-butyl-4-hydroxyanisole (BHA), and ascorbic acid (84.1, 85.2, and 88.1%, respectively). The other extracts had low or no scavenging activity (Table S1). Considering the total reducting power (TRP) assay, the moss extracts examined in this research showed no considerable activity compared to standard substances (Table S1). 

**Figure 1:**
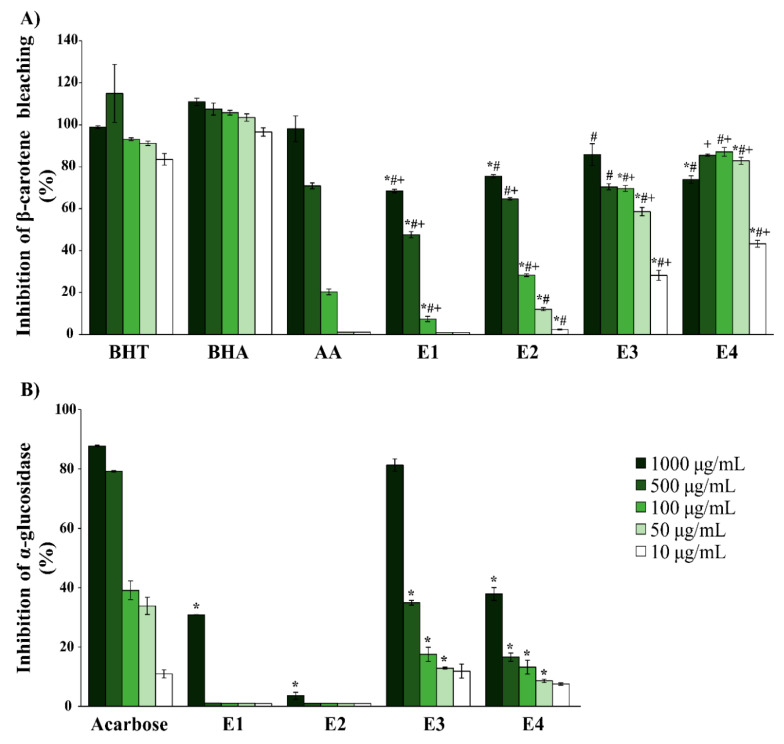
Biochemical analysis of *Hypnum cupressiforme* extracts as (**A**) antioxidant and (**B**) antidiabetic agents. The results are expressed as the mean ± standard error from an experiment performed in triplicate (#, +, * *p* < 0.05 different moss extracts vs. different standard substances. Symbols # and + were used for standards BHA and AA (ascorbic acid), while * was used for all the other standards).

The results of the β-carotene bleaching assay are shown in Figure 1A. According to the results, E3 and E4 extracts at the concentrations of 100, 50, and 10 µg/mL displayed significant β-carotene bleaching inhibitory activities compared with the same concentrations of standard substance ascorbic acid. The percentage of inhibition of these extracts was approximately four fold higher than the standard substance ascorbic acid. 

The activity of the same extracts at the concentrations of 1000 and 500 µg/mL was similar to the activity of ascorbic acid at the same concentrations. According to the results, E3 and E4 extracts have shown high inhibition percentage of β-carotene bleaching compared to ascorbic acid. In comparison with BHT and BHA, E3 and E4 extracts have shown moderate activity. Extracts E1 and E2 have shown low to moderate inhibitory activity compared with the standard substances.

#### 2.2.2. Evaluation of Antidiabetic Activity

Considering α-amylase inhibitory activity, the results have shown that the investigated extracts do not possess inhibitory activity against the α-amylase enzyme, compared to acarbose (Table S2). The results of α-glucosidase inhibitory activity (Figure 1B), however, have shown that the investigated extracts possess certain inhibitory potential against the α-glucosidase enzyme. The most active was the E3 extract, followed by the E4 extract. Both of the extracts have shown a similar percentage of inhibition to the standard substance at the concentration of 10 µg/mL. According to these results, the E3 extract has shown high inhibition of α-glucosidase, while the E4 extract exhibited moderate inhibition of this enzyme. Nevertheless, it is important to point out that both extracts displayed high inhibition properties at the lowest tested concentration (10 µg/mL).

#### 2.2.3. Evaluation of Antineurodegenerative Activity

**Figure 2:**
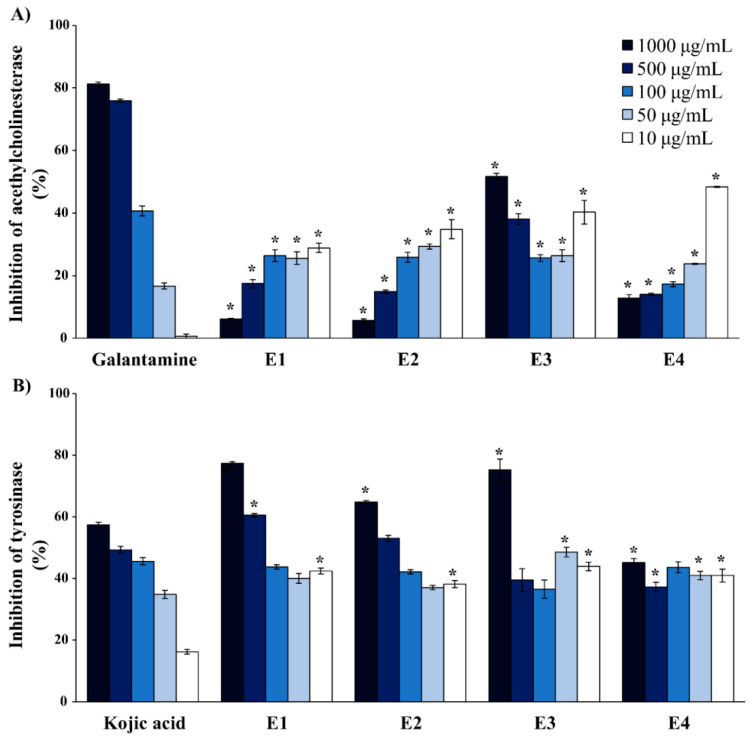
Biochemical analysis of *Hypnum cupressiforme* extracts as (**A**) antiacetylcholinesterase and (**B**) antityrosinase agents. The results are expressed as the mean ± standard error from an experiment performed in triplicate (* *p* < 0.05 different moss extracts vs. different standard substances).

The results for the inhibition of acetylcholinesterase (AChE) and tyrosinase are presented in Figure 2, respectively.

The highest AChE inhibition was achieved with E3 and E4, followed by E2 and E1 extracts. Although galantamine inhibited AChE in a concentration-dependent manner, the extracts performed the strongest activity at 10 µg/mL, except the E3 extract which performed the strongest activity at the highest tested concentration (1000 µg/mL), followed by the lowest tested concentration (10 µg/mL). Galantamine did not show inhibition of AChE at the concentration of 10 µg/mL, while the extracts at the same concentration inhibited the enzyme. According to the obtained results, extracts have shown significantly higher inhibition activity at lower concentrations, when compared to the standard substance. 

The evaluation of tyrosinase inhibitory effects showed that all four of the investigated moss extracts exhibit a high percentage of tyrosinase inhibition when compared to kojic acid. At the lowest applied concentration (10 µg/mL), all of the investigated extracts exhibited approximately 2.5-fold higher percentages of inhibition than kojic acid. Moreover, all investigated extracts at the remainingconcentrations (50, 100, 500, and 1000 µg/mL) exhibited similar or even higher inhibition activity than kojic acid at the same concentrations.

### 2.3. Immunomodulatory Activities of the Extracts

#### 2.3.1. Evaluation of Biocompatibility of *Hypnum cupressiforme* Extracts (MTT Assay)

The effect of investigated moss extracts on the proliferation potential of the normal human fibroblast cell line, MRC-5, during 24 h was measured by the 3-(4,5-dimethylthiazol-2-yl)-2,5-diphenyltetrazolium bromide (MTT) test. This test was used to estimate the biocompatibility of the extracts. The results have shown statistically significant biocompatibility of E1, E2, and E3 extracts (Table S3). Biocompatibility is expressed as a high percentage of viable normal human fibroblasts after the treatment with investigated moss extracts for 24 h. The viability of MRC-5 cells after the treatment with all moss extracts was higher than 85%. This biocompatibility analysis demonstrates that the tested extracts do not exhibit any nonspecific toxic effect to normal human fibroblast cells, thus qualifying them as suitable for further investigation in the in vitro model systems for the treatment of some pathological conditions in humans.

#### 2.3.2. Anti-Neuroinflammatory Potential of *Hypnum cupressiforme* Extracts

**Figure 3:**
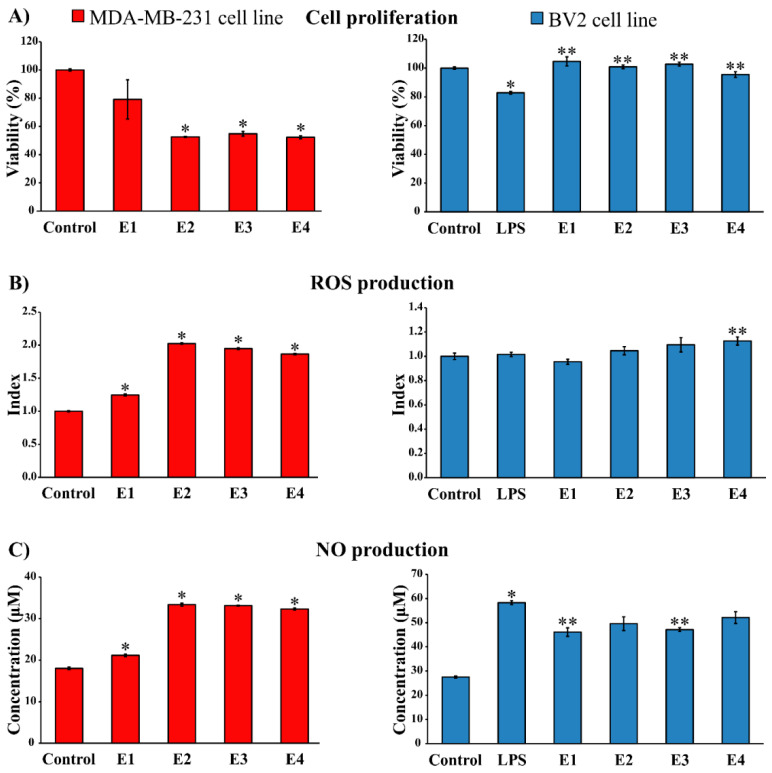
Immunomodulatory potential of *Hypnum cupressiforme* extracts (final concentration 10 µg/mL) on (**A**) cell proliferation, (**B**) ROS production, and (**C**) NO production. The results are expressed as mean ± standard error of a representative experiment performed in quadruplicate. (* *p* < 0.05 different moss extracts and LPS-stimulated control cells vs. non-stimulated control cells; ** *p* < 0.05 different moss extracts vs. only LPS-stimulated control cells).

Due to the good biocompatibility of the investigated extracts and potent anti-inflammatory activity of different moss extracts shown in the existing literature [1,15], the anti-neuroinflammatory activity of the investigated extracts against microglia cell line BV2 has been tested. The results are presented in Figure 3.

The viability of BV2 microglia cells was evaluated using MTT assay (Figure 3A, right). The results have shown that, compared with the control cells, lipopolysaccharide (LPS) significantly reduced the viability of BV2 cells. Furthermore, treatment with all investigated moss extracts has led to a significant recovery of the viability of LPS-stimulated BV2 cells.

The potential of all moss extracts to modulate reactive oxygen species (ROS) and nitric oxide (NO) production was further evaluated, as significant microglia inflammatory mechanisms. The treatment with moss extracts E1, E2, and E3 did not affect the production of superoxide anion radical (O_2_^−^) by BV2 cells, while E4 extract increased the production of ROS in these cells, compared to untreated cells (Figure 3B, right). 

The results presented in Figure 3C, right, show that *H. cupressiforme* extracts lead to a decrease in NO production of LPS-stimulated BV2 cells. Extracts E1 and E3 significantly inhibited NO production of LPS-treated BV2 cells measured by nitrite level in cell-free supernatants. This indicates that extracts E1 and E3 have the potential to suppress high NO production, which is an important inflammatory mechanism mediated by inflammatory microglia.

#### 2.3.3. Antiproliferative Potential of *Hypnum cupressiforme* Extracts

According to the good biocompatibility of the investigated extracts and potent antitumor activity of different moss extracts shown in the existing literature [6,16], the antiproliferative activity of the investigated extracts against cancer cell lines, HCT-116 and MDA-MB-231, has been tested.

The data presented in Figure 3A, left, shows that the investigated moss extracts exhibit potent and selective antiproliferative activity against human breast cancer cell line MDA-MB-231. Extracts E2, E3, and E4 showed statistically significant inhibition of proliferation compared to untreated cells (control). On the other hand, against human colon cancer cell line HCT-116, the tested moss extracts did not express an antiproliferative effect (Table S4). 

In the nitro blue tetrazolium (NBT) assay, the effects of *H. cupressiforme* extracts on superoxide anion radical (O_2_^−^) production by HCT-116 and MDA-MB-231 cells after 24 h have been evaluated. 

All of the investigated moss extracts have led to a significant increase in O_2_^−^ production by MDA-MB-231 cells, compared to the production of control cells (Figure 3B, left). Extracts E2, E3, and E4 displayed a higher increase in O_2_^−^ production than extract E1. However, the investigated extracts did not express any effect on ROS production by HCT-116 cells (Table S4).

Further antiproliferative potential was evaluated through the effect of extracts on nitrite concentration in supernatants of HCT-116 and MDA-MB-231 cells, tested by Griess assay. The concentration of nitrites was used as an indicator of the nitric oxide (NO) level in the investigated cell-free supernatants. 

Treatment with moss extracts significantly increased the production of NO by MDA-MB-231 cells, compared to the level of NO in supernatants of untreated cells (Figure 3C, left). As in the case of the impact of moss extracts on ROS production, the investigated extracts did not express an effect on NO production by HCT-116 cells (Table S4).

## 3. Discussion

In the present paper, the focus was on the chemical characterization as well as on antioxidant, antidiabetic, anti-neuroinflammatory/antineurodegenerative, and antitumor effects of four extracts (ethanolic, aqueous-ethanolic, ethyl acetate, and aqueous) of *H. cupressiforme* from Vršačke Planine Mts. Nature Park, Serbia.

The chemical characterization of moss extracts was performed using spectrophotometric assays (total phenolic, total phenolic acid, total flavonoid, total flavonol, total triterpenoid, and total coumarin contents) and liquid chromatography coupled to mass spectrometry (LC-MS). Literature data of the chemical composition of *H. cupressiforme* are scarce, reporting the presence of n-alkanes, triglycerides, and sterols [17]. Moreover, the isolation of several new to science flavonoid constituents (five new biflavonoids and a 3′-phenylaromadendrin) from this moss has been previously reported [17,18]. Based on the results of spectrophotometric assays, it can be concluded that the highest concentration of the investigated secondary metabolites of *H. cupressiforme* was obtained when ethyl acetate was used as solvent. A previous report [12] on the total phenolic content in *H. cupressiforme* ethanolic extract revealed a lower concentration of phenols than the onefound in the current study. The content of phenolic compounds in a certain plant depends on several factors such as substrate type; altitude; climate; as well as habitat, environmental condition, or geo-/geno-type [19], which could have influenced the differences in the obtained phenolic contents of the extracts. Using LC-MS analysis made it possible to identify 14 phenolic compounds in *H. cupressiforme* extracts. The major phenolic compounds present in this moss were kaempferol and five phenolic acids: *p*-hydroxybenzoic, protocatechuic, *p*-coumaric, gallic acid, and caffeic acid. These compounds were distributed unevenly among the investigated moss extracts, leading to the conclusion that they have different solubility in the used solvents. The presence of kaempferol in *H. cupressiforme* has already been reported earlier [18]. Some of the compounds identified in *H. cupressiforme* by LC-MS, including *p*-hydroxybenzoic, 5-*O*-caffeoylquinic, caffeic, *p*-coumaric, protocatechuic acids, and apigenin, were previously revealed in other moss species [9]. 

The chemical characterization of *H. cupressiforme* extracts presented in this study has shown that various phenolic acids, flavonoids, and terpenoids can be found in this moss. These compounds exhibit a range of biological activities and can be useful in treating various pathological conditions in humans [1,16], so further part of this research was based on the evaluation of antioxidant, antidiabetic, anti-neuroinflammatory/antineurodegenerative, and antitumor potential of the investigated extracts.

The examined moss extracts showed low radical scavenging activity compared to standard substances evaluated by the DPPH method. A previous study on radical scavenging activity demonstrated high antioxidant activity of *H. cupressiforme* methanolic extract [6]. This discrepancy in the obtained results could be explained by different extraction solvent and extraction procedure overall, different radical final concentration, or contrasting ecological conditions of the growth site such as season and climate [20]. For instance, it has been shown that the content of secondary metabolites in mosses depends on the season, with the greatest number of biochemical features manifested in the summer [21]. The probable explanation is that biological activities in mosses during summer are more intense than during other seasons, with more protective substances that help tolerate droughts. The moss samples examined in this study were collected during springtime, so it may be expected that they show different potential in terms of some biochemical features, such as radical scavenging activity. 

Regarding radical scavenging activity, it is known that the most effective compounds are phenols, which are capable of donating a hydrogen atom from their hydroxyl group [22]. Although mosses are rich in phenolic compounds, their radical scavenging activity seems to be lower than that in vascular plants. In vascular plants, the phenolic and flavonoid contents usually directly correlate with the radical scavenging capacity of the plant [23]. However, in mosses, there is no direct correlation between these terms [9]. This may be the reason why the investigated extracts did not exhibit high radical scavenging activity despite the presence of phenolic compounds in them. Furthermore, numerous triterpenoids have demonstrated significant radical scavenging activity [24]. The correlation between the triterpenoids in *H. cupressiforme* and its radical scavenging activity needs to be further investigated. 

It has been reported that anthocyanins and flavonols possess high activity in the β-carotene bleaching test, followed by flavanols and phenolic acids [25]. Some of the compounds that may express this activity are kaempferol, gallic acid, and caffeic acid [25], and they are identified in the investigated *H. cuppresiforme* extracts by LC-MS. Therefore, these compounds may contribute to the β-carotene bleaching inhibitory activity of the investigated moss. To the best of our knowledge, there is no previous report on the β-carotene bleaching activity of the investigated extracts of *H. cupressiforme*. The antioxidant activity obtained for ethyl acetate and aqueous extracts in the β-carotene bleaching method was significant, while the antioxidant activities measured by the DPPH and TRP methods were quite low. One of the main reasons for this result is the fact that these methods are based on different reaction mechanisms. The DPPH assay is based on a hydrogen radical donating to a certain substance, the TRP assay is based on electron transfer between the substrate and tested sample, while the β-carotene bleaching assay is based on spearing the already present antioxidant [26]. These results clearly demonstrate the significance of applying more than one analytical method when assessing the antioxidant activity of some new extracts. 

Diabetes mellitus (DM) is a chronic disease characterized by insulin deficiency and/or insulin insensitivity which leads to chronic hyperglycemia and disturbance of carbohydrate, lipid, and protein metabolism. One of the therapeutic approaches for DM therapy is inhibition of carbohydrate hydrolyzing enzymes, α-amylase and α-glucosidase. Although synthetic inhibitors of carbohydrate hydrolyzing enzymes are in clinical use, their prices are usually high and they have many side effects. Inhibitors isolated from medicinal plants are considered as an alternative due to fewer side effects and greater efficiency of these compounds. Phenolic compounds, especially flavonoids, phenolic acids, and tannins, apart from being effective antioxidants, have been reported as potent inhibitors of α-glucosidase and α-amylase [27]. The mechanism of this inhibition depends on the type of polyphenols, as well as their concentration. Monomeric polyphenols inhibit enzyme activity by blocking the catalytic sites, while polymeric polyphenols can form a non-digestible complex with the enzyme [28]. Some of the compounds previously reported to inhibit α-glucosidase and α-amylase are gallic, caffeic, 5-*O*-caffeoylquinic acids, kaempferol, quercetin, naringenin, and apigenin [29]. The presence of these compounds in the examined *H. cupressiforme* extracts, confirmed by LC-MS, could explain their significant inhibitory effect on α-glucosidase and α-amylase activity which was noticed in the study. Although studies have shown the antidiabetic potential of bryophytes [1], no previous report has been given on the antidiabetic potential of the *H. cupressiforme*. It has been reported that an ideal inhibitor of α-amylase and α-glucosidase could exhibit specificity for the respective enzyme, effectively reducing the postprandial hyperglycemia, without showing cytotoxic activity to target cells. Furthermore, a better effect could be achieved if the examined compound would mildly inhibit α-amylase and strongly inhibit α-glucosidase [30]. The investigated extracts of *H. cupressiforme* have demonstrated these properties and therefore could be considered for potential therapeutic application to delay postprandial hyperglycemia, although further in vivo research is required.

The inhibitory potential of the investigated moss extracts against AChE and tyrosinase, which are associated with the development of various neurodegenerative disorders that are mostly mediated by neuroinflammation, was evaluated. AChE is an enzyme that catalyzes the breakdown of a neurotransmitter called acetylcholine, thus regulating its levels in the synapses. One of the main characteristics of Alzheimer’s disease (AD) is memory loss caused by reduced levels of acetylcholine. Therefore, the inhibition of AChE is an effective therapeutic approach in treating AD [31]. On the other hand, tyrosinase is a key enzyme involved in the production of melanin in both skin and hair and may contribute to neuromelanin formation in the central nervous system. The production and accumulation of neuromelanin and the damage of neurons associated with Parkinson’s disease (PD) have been extensively studied [32]. The inhibition of tyrosinase has become a prominent target in drug development and research of PD. Due to high prices and the side effects of current treatment strategies for neurodegenerative diseases, there is an increased need for the development of novel therapeutic strategies [33]. 

This is the first report on the antineurodegenerative activity of the *H. cupressiforme*. An opposite dose–response observed in the AChE inhibition assay may be caused by mutual interaction between compounds at higher concentrations, which could prevent their inhibitory potential. Moreover, studies have shown that some extracts show the greatest AChE inhibitory potential at lower concentrations when occupying only a small number of AChE receptors [34]. The majority of phytochemicals with AChE and tyrosinase inhibitory potential belong to flavonoids, alkaloids, and terpenoids [35]. Some of the flavonoids identified in the examined *H. cupressiforme* extracts, such as naringenin, apigenin, kaempferol, and quercetin, were previously reported to possess AChE and tyrosinase inhibitory potential [36]. In addition, some of the phenolic acids present in investigated extracts, such as *p*-hydroxybenzoic, protocatechuic, *p*-coumaric, and caffeic acids, have been investigated for their tyrosinase inhibition potential [37]. 

Microglia represent a specialized population of macrophages extensively distributed in the brain. They mediate immune responses in the central nervous system and remove damaged neurons and cellular debris through the process of phagocytosis [38]. When microglia are activated, in response to pathogens or brain injury, they can enhance neuroinflammation by secreting various proinflammatory mediators, including NO and ROS. These molecules are associated with neurodegenerative diseases such as AD, PD, multiple sclerosis, and cerebral ischemia [39]. Therefore, the reduction of proinflammatory mediators production by microglia is considered to be an essential therapeutic strategy for the prevention or treatment of these disorders [38].

As the antineurodegenerative effect of analyzed extracts was shown, the survey of the anti-neuroinflammatory potential of the extracts on LPS-stimulated BV2 microglial cells was performed. The obtained results suggest that *H. cupressiforme* extracts possess the ability to diminish the production of NO by activated microglia that participate in the majority of neurodegenerative diseases. Inducible nitric oxide synthase (iNOS) is an enzyme that catalyzes the production of NO. Various compounds from plants have been proven to inhibit the expression of iNOS in LPS-activated macrophages. Most of the herbal medicines reduce iNOS-mediated NO production through the inactivation of nuclear factor-kappa B (NF-κB) [40]. It has been reported that flavonoids exhibit strong inhibitory activity on LPS-induced NO production [41]. Kaempferol, quercetin, and naringenin were among the most efficient flavonoids regarding the inhibition of NO production. Additionally, caffeic acid has also shown potent inhibitory activity on NO production [41]. Some of these compounds, identified in *H. cupressiforme* extracts by LC-MS, may contribute to the anti-neuroinflammatory activity of this moss. Altogether these anti-neuroinflammatory findings with considerable AChE and tyrosinase inhibitory potentials of these moss extracts highly suggest that the extracts of *H. cupressiforme* may be potential therapeutic candidates for the prevention and treatment of neurodegenerative disorders, although further investigation is necessary to confirm their anti-inflammatory activities. 

Antitumor agents are one of the most important research topics in drug development. The successful treatment of several tumor types is still a challenge and there are many research studies focused on finding new therapies with fewer side effects. Plants continue to play a major role in drug discovery due to their antiproliferative and proapoptotic properties [42]. Various studies have reported significant cytotoxic activity in the extracts of bryophytes [15,16]. The antitumor activity in the investigated extracts was tested as their ability to inhibit the proliferation of two tumor cell lines, HCT-116 and MDA-MB-231. All of the investigated moss extracts, except ethanolic, exerted strong antiproliferative activity against MDA-MB-231 cells, with an inhibition rate of approximately 50%. On the other hand, there was no significant antiproliferative effect on HCT-116 cells. Based on these results, it can be concluded that *H. cupressiforme* extracts exhibit selectivity in the inhibition of the cell cycle between different tumor cells. This is in line with literature data, where it can be seen that *H. cupressiforme* methanolic extracts exhibit a strong antiproliferative effect on HeLa cancer cells and a moderate antiproliferative effect on lung cancer A549 cells [6]. An extensive literature survey showed that there is no previous report on the antiproliferative activity against the MDA-MB-231 cell line of *H. cupressiforme*.

Phenolic compounds and terpenoids possess the ability to prevent tumor cell progression by different antitumor mechanisms [16,43]. Generally, these compounds can modulate the redox status and act on cellular processes such as cell proliferation, differentiation, inflammation, apoptosis, and angiogenesis. They have the ability to regulate cell signal transduction and gene expression, thus controlling tumor development [43]. Flavonoids, among many other compounds, have been investigated for potential antitumor activity. In human breast cancer cell lines, such as MDA-MB-231, flavonoids have been reported to downregulate the expression of mutant p53 protein to almost undetectable levels and to inhibit the production of heat shock proteins [44]. Furthermore, it has been experimentally proven that flavonoids apigenin, quercetin, kaempferol, and quercetin 3-*O*-rutinoside (rutin) are potent inhibitors of the transcription factor NF-κB, responsible for the activation of many genes involved in cell proliferation, thus acting as antiproliferative agents [44]. Given that the moss extracts investigated in this study contain certain amounts of phenolic compounds and terpenoids, these compounds might contribute to a significant antiproliferative activity of all extracts, except the ethanolic one. 

ROS and NO are essential for maintaining cell homeostasis, but can also be involved in the development of different pathologies. The roles of ROS and NO in cancer cells are controversial, with some reports indicating their antitumor potential, while others suggest they have a role in tumor promotion [45,46]. Tumor cells generally demonstrate a constant increase in the generation of ROS, which in turn makes these cells more vulnerable to further oxidative stress. This biochemical characteristic has been used to selectively kill tumor cells by further elevation of cellular ROS. Increased ROS production in tumor cells may induce cell death through the induction of apoptosis via death signaling pathways [45]. NO was reported to inhibit cell proliferation and induce cell death by affecting apoptosis-related mitochondrial proteins [47]. It was shown that breast cancer cells treated with various apoptotic agents have shown an increased production of NO [48]. All four extracts tested in this study have led to a significant increase in ROS production by breast cancer MDA-MB-231 cells. All examined extracts significantly increased the production of NO by MDA-MB-231 cells, as well. The significant antiproliferative potential of *H. cupressiforme* extracts demonstrated against MDA-MB-231 cells could be mediated via increased ROS and NO production. However, the exact mechanism remains to be further explored.

## 4. Materials and Methods

### 4.1. Reagents and Standards

Acarbose, acetylcholine iodide, and acetylcholinesterase from *Electrophorus electricus* Linnaeus, 1766, and aluminum chloride (AlCl_3_), ascorbic acid, BHA (2-tert-butyl-4-hydroxyanisole), BHT (3,5-di-tert-butyl-4-hydroxytoluene), protocatechuic acid, 5-*O*-caffeoylquinic acid, *p*-hydroxybenzoic acid, caffeic acid, *p*-coumaric acid, quercetin 3-*O*-rutinoside, quercetin 3-*O*-glucoside, isorhamnetin 3-*O*-glucoside, eriodictyol, apigenin, naringenin, kaempferol, acacetin, coumarin, disodium hydrogen phosphate dodecahydrate, DMSO (dimethyl sulfoxide), DPPH (2,2-diphenyl-1-picrylhydrazyl), DTNB (5,5′-dithio-bis(2-nitrobenzoic acid)), Folin–Ciocalteu reagent, galantamine natural for system suitability, gallic acid, iron(III) chloride (FeCl_3_), kojic acid, L-DOPA (3,4-dihydroxy-l-phenylalanine), LPS (lipopolysaccharide), Lugol’s solution, MTT (3-(4,5-dimethylthiazol-2-yl)-2,5-diphenyltetrazolium bromide), pNPG (4-nitrophenyl β-d-glucopyranoside), phosphoric acid, potassium dihydrogen phosphate, quercetin, sodium acetate (CH_3_COONa), sodium bicarbonate (NaHCO_3_), sodium carbonate anhydrous (Na_2_CO_3_), sodium chloride (NaCl), sodium dihydrogen phosphate, sodium hydrogen phosphate, sodium nitrite (NaNO_2_), sodium phosphate monobasic dihydrate, sulfanilamide, sulfanilic acid, Trizma base, tyrosinase from *Agaricus bisporus* (J.E. Lange) Imbach, ursolic acid, vanillin, α-amylase, α-glucosidase (from *Saccharomyces cerevisiae* Meyen ex E.C. Hansen) type I, and β-carotene were purchased from Sigma-Aldrich, St. Louis, MO, USA. Chloroform, ethanol, glacial acetic acid and hydrochloric acid were obtained from Zorka Pharma, Šabac, Serbia. Sodium molybdate dihydrate (Na_2_MoO_4_·2H_2_O) was obtained from Dispo-chem, Romsey, UK. Sodium hydroxide (NaOH) was purchased from NRK inženjering, Belgrade, Serbia. Aluminum nitrate nonahydrate (Al(NO_3_)_3_·9H_2_O) and potassium acetate (CH_3_COOK) were obtained from Carlo Erba Reagents, Barcelona, Spain. Dipotassium phosphate, methanol, and perchloric acid were purchased from VWR, Radnor, PA, USA. Lead acetate trihydrate (Pb(C_2_H_3_O_2_)_2_·3(H_2_O)), potassium ferricyanide(III), and trichloroacetic acid were obtained from Superlab, Belgrade, Serbia. Linoleic acid and Tween 40 were purchased from Acros Organics, Geel, Belgium, while 1% starch solution was purchased from Carl Roth, Karlsruhe, Germany. DMEM (Dulbecco’s Modified Eagle Medium), FBS (Fetal Bovine Serum), and PBS (Phosphate-Buffered Saline) were obtained from GIBCO, Invitrogen, Carlsbad, CA, USA. NBT (Nitro Blue Tetrazolium) was obtained from SERVA, Heidelberg, Germany.

### 4.2. Plant Material

#### 4.2.1. Moss Material Collection

**Figure 4:**
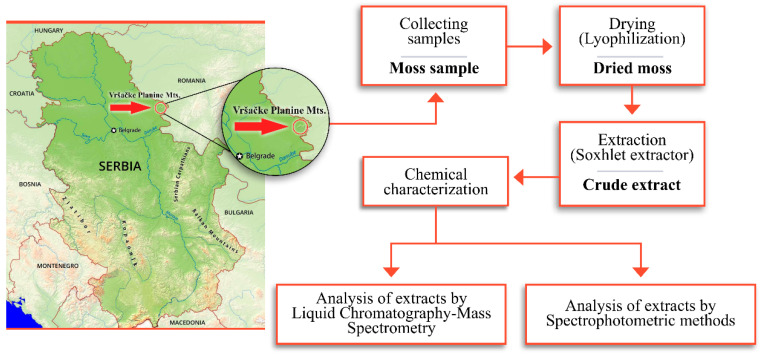
The position of the investigated collection area (Vršačke Planine Mts.) in Serbia with illustrated steps in the preparation and characterization of the extracts.

Moss material was collected in Vršačke Planine Mts. (Serbia) (N45.128208, E21329945, 370 m a.s.l.), from the siliceous rock outcrops within the forest openings in May 2019 (leg./det. M. S. Sabovljevic and A. D. Sabovljevic, 11th May 2019; voucher BEOU bryo collection *s*/*n*). Mosses were placed in paper bags and kept at room temperature. The room-dried and cleaned material (i.e., green tips with no older parts and substrate remnants) was then lyophilized and ready for extraction. The whole study flow (except biological evaluation) is presented in Figure 4.

#### 4.2.2. Preparation of the Extracts

The plant material was dried, and the extraction was performed with different solvents in the Soxhlet apparatus for 10 h. Four different solvents were used: 96% ethanol, a mixture of water and ethanol in 1:1 ratio, ethyl acetate, and water. The extracts were labeled as follows; E1 for ethanolic extract, E2 for aqueous-ethanolic extract, E3 for ethyl acetate extract, and E4 for aqueous extract. The extracts were concentrated in vacuum (Buchi R-210 Rotavapor System, Marshall Scientific, Hampton, NH, USA) and stored at + 4 °C.

### 4.3. Chemical Characterization

#### 4.3.1. Total Phenolic Content

Total Phenolic Content (TPC) of the extracts was determined using the Folin–Ciocalteau method [49]. Briefly, 20 µL of each extract (1 mg/mL) was mixed with 100 µL of 10% Folin–Ciocalteu reagent and kept for 6 min at room temperature. After that, 80 µL of 7.5% sodium carbonate anhydrous was added to the reaction mixture and kept for 120 min in the dark at room temperature. The absorbance was measured at 740 nm, using Multiskan Sky Thermo Scientific microtiter plate reader, Vantaa, Finland. The negative control contained distilled water instead of the sample. TPC was calculated from the curve equation of gallic acid and the results were expressed as milligrams of gallic acid equivalents per gram of dry extract (mg GAE/g dry extract). 

#### 4.3.2. Total Phenolic Acid Content

Total Phenolic Acid Content (TPAC) of the extracts was determined using a modified procedure [50]. For the determination of TPAC in extracts, 10 µL of extract (1 mg/mL) was mixed with 20 µL of Arnow reagent (10% *w*/*v* of sodium molybdate and 10% *w*/*v* sodium nitrite), 20 µL 0.1 M hydrochloric acid, and 20 µL 1 M sodium hydroxide. After adding 100 µL of distilled water to the obtained mixture, the absorbance was measured immediately at 490 nm, using Multiskan Sky Thermo Scientific microtiter plate reader, Vantaa, Finland. The control contained 50% ethanol instead of the sample. TPAC was calculated from the curve equation of caffeic acid in 50% ethanol. The results were expressed as milligrams of caffeic acid equivalents per gram of dry extract (mg CAE/g dry extract). 

#### 4.3.3. Total Flavonoid Content

Determination of Total Flavonoid Content (TFC) was performed by spectrophotometric method [51]. Each well was filled with 50 µL of extracts (1 mg/mL), 205 µL 80% ethanol, 5 µL 10% aluminum nitrate nonahydrate, and 5 µL 1 M potassium acetate solution. After 40 min of incubation at room temperature, the absorbance was measured at 415 nm, using Multiskan Sky Thermo Scientific microtiter plate reader, Vantaa, Finland. The negative control contained 96% ethanol instead of the sample. The calibration curve for TFC was made using a quercetin standard solution under the same procedure as earlier described. TFC was calculated from the curve equation of quercetin and the results were expressed as milligrams of quercetin equivalents per gram of dry extract (mg QE/g dry extract). 

#### 4.3.4. Total Flavonol Content

The Total Flavonol Content (TFlC) of the extracts was determined using a modified procedure [50]. Briefly, 40 µL of methanolic solutions of extracts (1 mg/mL) was mixed with the same volume of aluminum chloride (20 mg/mL) solution in methanol and 120 µL (50 mg/mL) methanolic solution of sodium acetate. The absorbance of the resulting yellow complex at 440 nm was read after 2.5 h, using Multiskan Sky Thermo Scientific microtiter plate reader, Vantaa, Finland. The negative control contained 100% methanol instead of the sample. Series of quercetin in 100% methanol were prepared for the calibration curve. TFlC was calculated from the curve equation of quercetin and the results were expressed as milligrams of quercetin equivalents per gram of dry extract (mg QE/g dry extract). 

#### 4.3.5. Total Triterpenoid Content

Total Triterpenoid Content (TTC) was determined according to a previously reported method [52]. Briefly, all extracts were individually dissolved in 100% methanol at concentrations 1 and 10 mg/mL. Then, 10 µL of each of these sample solutions was mixed with 15 µL of vanillin-glacial acetic acid solution (5% *w*/*v*) and 50 µL of perchloric acid solution. The sample solutions were incubated for 45 min at 60 °C and then cooled to the ambient temperature. The negative control contained 100% methanol instead of the sample. After the addition of glacial acetic acid (225 µL), each sample solution’s absorbance was measured at 548 nm, using Multiskan Sky Thermo Scientific microtiter plate reader, Vantaa, Finland. Ursolic acid dissolved in 100% methanol and was used for making the calibration curve. TTC was calculated from the curve equation of ursolic acid, and the results were expressed as milligrams of ursolic acid equivalents per gram of dry extract (mg UAE/g dry extract). 

#### 4.3.6. Total Coumarin Content

Total Coumarin Content (TCC) of the extracts was performed according to a slightly modified procedure [53]. Each well was filled with 2 µL of extract dissolved in 80% methanol (10 mg/mL), 8 µL of distilled water, and 2 µL of lead-acetate solution (5% *w*/*v*). Another 28 µL of distilled water was added to each well, followed by 160 µL of hydrochloric acid (0.1 M). The reaction mixture was incubated for 30 min at ambient temperature. The absorbance was measured at 320 nm, using Multiskan Sky Thermo Scientific microtiter plate reader, Vantaa, Finland. The negative control contained 100% methanol instead of the sample. Coumarin dissolved in methanol was used for the calibration curve. TCC was calculated from the curve equation of coumarin and the results were expressed as milligrams of coumarin equivalents per gram of the dry extract (mg CE/g dry extract). 

#### 4.3.7. Liquid Chromatography–Mass Spectrometry

Separation of compounds of interest was performed using a Dionex Ultimate 3000 UHPLC system equipped with a diode array detector (DAD) that was connected to TSQ Quantum Access Max triple-quadrupole mass spectrometer equipped with heated electrospray ionization probe (HESI-II, ThermoFisher Scientific, Bremen, Germany) in negative ionization mode.

A Syncronis C18 column (100 × 2.1 mm, 1.7 µm particle size) at 40 °C was used for compound separation: Flow rate was set to 0.3 mL/min and the mobile phase consisted of (A) water + 0.1% formic acid and (B) acetonitrile. Linear gradient program was used as follows; 0.0–1.0 min 5% B, 1.0–14.0 min from 5% to 95% (B), 14.0–14.1 min from 95% to 5% (B), and 5% (B) for 6 min.

The parameters of the ion source and the other MS data necessary for quantification were as previously described in the literature [54]. ThermoFisher Scientific Xcalibur software (version 2.1) was used for instrument control, data acquisition, and data analysis.

### 4.4. Biochemical Assays

#### 4.4.1. DPPH Assay

The scavenging activity of moss extracts was evaluated using DPPH assay [55] with slight modifications. Briefly, 20 µL of sample solutions in appropriate solvents (concentrations of 10, 50, 100, 500, and 1000 µg/mL) and 180 µL of fresh methanolic solution of DPPH (40 µg/mL) were added to each well of a microtiter plate. Methanol dissolved in DPPH solution was used as a negative control. Butylated hydroxytoluene (BHT), butylated hydroxyanisole (BHA), and ascorbic acid were used as positive controls (standards). The absorbance of the reaction mixture was measured after 30 min in the dark at room temperature at 517 nm, using Multiskan Sky Thermo Scientific Microtiter plate reader, Vantaa, Finland. 

The decrease of absorption of DPPH radical at 517 nm was calculated using the following equation,

Inhibition of DPPH radical (%) = [(*Ac* − *As*)/*Ac*] × 100 (1)

where *Ac* represents the absorbance of the negative control and *As* represents the absorbance of the test samples at different concentrations.

#### 4.4.2. Total Reducing Power Assay

The ability of the extracts to reduce iron(III) was assessed by the slightly modified method [56], and following a previous procedure [57]. Briefly, 20 µL of each extract in an appropriate solvent (concentrations of 10, 50, 100, 500, and 1000 µg/mL) was mixed with 40 µL of phosphate buffer (0.2 M, pH 6.6) and 40 µL of 1% potassium ferricyanide(III) solution. The mixture was incubated for 20 min at 45 °C, after which 40 µL of trichloroacetic acid (10%, *w*/*v*), 40 µL of distilled water, and 8 µL of 0.1% iron(III) chloride were added. After an incubation of 10 min at room temperature, the absorbance was measured at 700 nm minutes, using Multiskan Sky Thermo Scientific, Vantaa, Finland microtiter plate reader. The negative control was prepared in the same manner as the reaction mixture, with 20 µL of appropriate solvent instead of the sample. BHT, BHA, and ascorbic acid were used as positive controls (standards) and data are presented only against ascorbic acid as the same results were obtained. The Total Reducing Power (TRP) of the samples is expressed as µmol of Ascorbic Acid Equivalents (AAE) per gram of dry extract (µmol AAE/g dry extract). 

#### 4.4.3. β-Carotene Bleaching Assay

β-carotene bleaching assay was performed according to a slightly modified procedure [58]. The emulsion was prepared by adding linoleic acid (6.25 µL) and Tween 40 (50 mg) into a solution of β-carotene in chloroform (125 µL, 4 mg/mL). Moreover, 125 µL of chloroform was added to the prepared emulsion. Chloroform was removed using a rotary evaporator (Buchi rotavapor R-114, Marshall Scientific, Hampton, NH, USA) at 40 °C, after which 25 mL of distilled water was added with vigorous shaking. The solutions of samples (concentrations of 10, 50, 100, 500, and 1000 µg/mL) and standards BHT, BHA, and ascorbic acid were prepared in appropriate solvents. Afterward, 200 µL of emulsion and 28 µL of the test substance (extracts, standards, 100% methanol – as negative control) were mixed. The absorbance was measured immediately (t_0_ = 0 min) and after 2 h of incubation (t_120_ = 120 min) at 490 nm, using Multiskan Sky Thermo Scientific Microtiter plate reader, Vantaa, Finland. 

The antioxidant activity of the samples was evaluated in terms of inhibition of β-carotene bleaching using the following equation,

Inhibition of β-carotene bleaching (%) = [(*A*_120_ − *C*_120_)/(*C*_0_ − *C*_120_)] × 100 (2)

where *A*_120_ and *C*_120_ symbolize the absorbances measured after 120 min for the sample and negative control, respectively, while *C*_0_ symbolizes the absorbance of the negative control immediately after the addition of all the reaction components.

#### 4.4.4. α-Amylase Inhibition Assay

In vitro determination of α-amylase inhibition activity was performed using the slightly modified Caraway–Somogyi iodine/potassium iodide method, according to Zengin et al. [59]. In brief, 25 µL of properly diluted extracts (concentrations of 10, 50, 100, 500, and 1000 µg/mL) were mixed with 50 µL of 0.5 mg/mL α-amylase enzyme solution. The solutions were prepared by dissolving the extracts and enzyme in sodium phosphate buffer (0.1 M, pH 6.8 with 6 mM sodium chloride). After 10 min of pre-incubation at 37 °C, 50 µL of 0.2% starch dissolved in phosphate buffer was added, and incubation continued for another 10 min at 37 °C. After that, 25 µL of 1 M hydrochloric acid was added to terminate the reaction, and 100 µL of Lugol’s solution was added to visualize the reaction. The absorbance was measured at 630 nm, using Multiskan Sky Thermo Scientific, Vantaa, Finland microtiter plate reader. Acarbose was used as a positive control (standard).

The percentage inhibition of α-amylase enzyme activity was calculated according to the following equation,

Inhibition of α-amylase (%) = [(*As* − *Ac*_1_)/*Ac*_2_] × 100 (3)

where *As* represents the absorbance of the reaction mixture with the test sample, *Ac*_1_ is the absorbance of enzyme control (contained buffer instead of the sample), and *Ac*_2_ is the absorbance of substrate control (contained buffer instead of enzyme).

#### 4.4.5. α-Glucosidase Inhibition Assay

Determination of α-glucosidase inhibitory activity was performed according to a previously reported method [60]. Briefly, 120 µL of extract (concentrations of 10, 50, 100, 500, and 1000 µg/mL) and 20 µL of enzyme solution (0.5 units/mL) in potassium phosphate buffer (0.1 M, pH 6.8) were added to microtiter plate and pre-incubated for 5 min at 37 °C. Moreover, 20 µL of 5 mM pNPG as a substrate was added to the mixture and incubation continued for another 20 min at 37 °C. Finally, the reaction was stopped by adding 80 µL of 0.2 M sodium carbonate anhydrous dissolved in a buffer, and the absorbance was measured at 405 nm, using Multiskan Sky Thermo Scientific microtiter plate reader, Vantaa, Finland. Acarbose was used as a positive control (standard). 

The percentage of α-glucosidase activity inhibition was calculated according to the following equation,

Inhibition of α-glucosidase (%) = [(*Ac* − *As*)/*Ac*] × 100 (4)

where *Ac* stands for the absorbance of the negative control (contained buffer instead of the sample), while *As* represents the absorbance of the reaction mixture with the test sample.

#### 4.4.6. Acetylcholinesterase Inhibitory Activity Assay

Acetylcholinesterase (AChE) inhibitory activity assay was performed according to the slightly modified method [61]. The test reaction mixture was prepared by adding 140 µL of sodium phosphate buffer (0.1 M, pH 7.0), 20 µL of DTNB, 20 µL of sample dissolved in buffer containing 5% DMSO (concentrations of 10, 50, 100, 500, and 1000 µg/mL), and 20 µL of AChE solution (5 units/mL) in Tris-HCl buffer (20 mM, pH 7.5). The negative control mixture contained sodium phosphate buffer instead of a sample. After incubation (15 min, 25 °C), the colorimetric reaction was initiated with the addition of 10 µL of acetylcholine iodide and the absorbance was measured at a wavelength of 412 nm, using a Multiskan Sky Thermo Scientific, Vantaa, Finland microtiter plate reader. Galantamine was used as a positive control (standard). 

The percentage of inhibition of AChE by the sample was determined using the formula,

Inhibition of AChE (%) = [(*Ac* − *As*)/*Ac*] × 100 (5)

where *Ac* symbolizes the absorbance of the negative control, while *As* represents the absorbance of the test sample.

#### 4.4.7. Tyrosinase Inhibitory Activity Assay

Tyrosinase inhibitory activity assay was performed according to a slightly modified method [62] using 96-well plates. The test reaction mixture was prepared by adding 80 µL of sodium phosphate buffer (0.1 M, pH 7), 40 µL of tyrosinase solution (46 units/L), and 40 µL of the sample (concentrations of 10, 50, 100, 500, and 1000 µg/mL). After adding 40 µL of L-DOPA in buffer and incubation (30 min, 25 °C), the absorbances were measured at 475 nm, using Multiskan Sky Thermo Scientific, Vantaa, Finland microtiter plate reader. The negative control contained sodium buffer instead of the sample. Kojic acid was used as a positive control (standard). 

The percentage of inhibition of tyrosinase activity was determined using the following formula,

Inhibition of tyrosinase (%) = [(*Ac* − *As*)/*Ac*] × 100 (6)

where *Ac* stands for the absorbance of the negative control and *As* stands for the absorbance of the test sample.

### 4.5. Biological Assays

#### 4.5.1. Cell Culture

Human embryonic lung fibroblast cell line MRC-5, murine microglial cell line BV2, human colon cancer cell line HCT-116, and human breast cancer cell line MDA-MB-231 were used in this study. All cells were obtained from American Tissue Culture Collection (ATCC, Manassas, VA, USA).

All cells were cultivated in DMEM and supplemented with 10% FBS, 1% glucose, and 1% antibiotics (penicillin and streptomycin). The cells were maintained at 37 °C in a humidified atmosphere containing 5% CO_2_. Near-confluent cells (100 µL) were seeded in 96-well microplate (10,000 per well for MRC-5, BV2, and MDA-MB-231 cells; 50,000 per well for HCT-116 cells). After 24 h of cell incubation, 100 µL of medium containing adequate investigated extract (50 µL of medium + 50 µL of extract) was added in each well of the microplate. For BV2 cells, 100 µL of LPS containing the investigated extracts (50 µL of LPS 1 µg/mL culture + 50 µL of extract) was added in each well. The incubation was continued for an additional 24 h. Untreated cells were used as control. The treatment concentration (10 µg/mL) was obtained by serial dilution of the stock solution, thus DMSO concentration decreased continuously. 

#### 4.5.2. Determination of Cell Proliferation/Metabolic Viability (MTT Assay)

The antiproliferative properties of the prepared extracts were tested utilizing the MTT assay [63] after 24 h of treatment. This colorimetric assay is based on the reduction of a yellow tetrazolium salt to formazan, an insoluble crystalline product with a deep purple color. Viable cells contain NAD(P)H-dependent oxidoreductase enzymes which are capable of this reduction.

After 24 h of cell incubation with extracts, 100 µL of medium was removed, and 10 µL of MTT solution (5 mg/mL final concentration in PBS) was added to each well and incubated at 37 °C in 5% CO_2_ for 3 h. The produced formazan was dissolved by overnight incubation by adding 100 µL of SDS-HCl (10% SDS in 0.1% 1N hydrochloric acid). Finally, the reduced MTT was assayed at 540 nm using a microplate reader (LKB 5060–006, LKB Instruments, Vienna, Austria). The results are expressed as the percentage of viable cells, calculated as the ratio between the absorbance of treated cells and the absorbance of the untreated control multiplied by 100.

#### 4.5.3. Determination of Superoxide Anion Radical (NBT Assay)

The concentration of superoxide anion radical (O_2_^−^) in the sample was determined by the NBT assay [64]. Nitro Blue Tetrazolium (NBT) undergoes reduction by O_2_^−^ to form diformazan, a dark blue insoluble precipitate. Levels of O_2_^−^ generated by the tissue can be quantified by measuring the absorbance of blue formazan. 

After 24 h of cell incubation with extracts, 100 µL of medium was removed, and 10 µL of NBT solution (5 mg/mL in PBS) was added to each well, followed by the cell incubation for 3 h at 37 °C in 5% CO_2_. To quantify the formazan production, formazan was solubilized in 100 µL SDS-HCl (10% SDS in 0.1% 1 N hydrochloric acid). The results are expressed as NBT index, calculated as the ratio between the absorbance of treated cells and the absorbance of the untreated control.

#### 4.5.4. Determination of Nitrites Level in Supernatants (Griess Assay)

The determination of nitrites (NO_2_^−^) as an indicator of NO level was performed using the spectrophotometric method [65]. The Griess reagent consists of two components: *N*-(1-naphthyl) ethylenediamine dihydrochloride in distilled water and 1% sulfanilamide (or sulfanilic acid) in 5% phosphoric acid. Equal volumes of the two components were mixed together to form the Griess reagent immediately prior to their application on the plate. The Griess reaction is based on the two-step reaction in which acidified NO_2_^−^ produces a nitrosating agent which subsequently reacts with sulfanilic acid to produce the diazonium ion. This ion is later coupled to *N*-(1-naphthyl) ethylenediamine to form the chromophoric azo-derivative which absorbs light at 540 nm.

After 24 h of cell incubation with extracts, 50 µL of medium from each well was transferred to an empty microplate. After that, 50 µL of prepared Griess reagent was added to each well. After 10 min incubation in the dark, the absorbance was measured at 540 nm using a microplate reader (LKB 5060–006, LKB Instruments, Vienna, Austria). The concentration of nitrites was calculated from the standard curve for nitrite and expressed in µmol/L (µM).

### 4.6. Statistical Analysis

Statistical analysis was performed using SPSS (IBM SPSS Statistics for Windows, Version 25.0., IBM Corporation, Armonk, NY, USA). Statistical evaluation was carried out by Independent Samples *t*-test. For all comparisons, *p* < 0.05 for control vs. extract was considered significant. All measurements were performed at least in triplicate and values are expressed as mean ± standard error.

## 5. Conclusions

In this study, four extracts of moss *H. cupressiforme* from the Vršačke Planine Mts., Serbia were evaluated for their potential antioxidant, antidiabetic, anti-neuroinflammatory/antineurodegenerative, and antitumor activities. The phytochemical analysis of the extracts has shown the presence of biologically active compounds such as flavonoids, phenolic acids, and triterpenoids distributed unevenly among the extracts. The investigated extracts have shown high antioxidant activity regarding the prevention of β-carotene bleaching, high tyrosinase inhibitory effect, and high α-glucosidase and AChE inhibitory activities at lower tested concentrations. The most potent were ethyl acetate and aqueous extracts. Additionally, ethanolic and ethyl acetate extracts manifested anti-inflammatory potential by reducing the production of NO by LPS-stimulated BV2 cells. All extracts, except the ethanolic one, have shown significant antiproliferative potential against MDA-MB-231 cancer cells, and also satisfying biocompatibility with normal cells. 

Based on the presented data, it can be concluded that moss *H. cupressiforme* from the Vršačke Planine Mts. (Serbia) is a promising candidate that may be useful in the prevention or treatment of various pathological conditions such as diabetes, Alzheimer’s disease, Parkinson’s disease, and breast cancer. Further in vitro and in vivo studies should focus on the mechanisms underlying the observed activities, all with the aim of developing new, more effective, green-factory made, and less toxic drugs for potential application in humans.

## Figures and Tables

**Table 1:** Extraction yield for *Hypnum cupressiforme* extracts.

Label	Solvent	Moss Weight (g)	Extract Weight (g)	Yield (%)
E1	Ethanol (96%)	10	0.42	4.2
E2	Water-ethanol (1:1, vol%)	10	0.80	8.0
E3	Ethyl-acetate	10	0.06	0.6
E4	Water	7.6	2.00	26.3

**Table 2:** Chemical characterization of *Hypnum cupressiforme* extracts.

Samples	TPC (mg GAE/g Extract)	TPAC (mg CAE/g Extract)	TFC (mg QE/g Extract)	TFlC (mg QE/g Extract)	TTC (mg UAE/g Extract)
E1	6.25 ± 0.48	67.41 ± 6.97	35.00 ± 1.34	ND ^1^	88.37 ± 1.55
E2	7.38 ± 0.34	7.08 ± 2.36	12.43 ± 0.49	ND	75.93 ± 2.97
E3	15.33 ± 0.95	339.93 ± 14.03	58.86 ± 2.82	14.11 ± 1.33	235.95 ± 4.09
E4	18.21 ± 0.73	8.31 ± 3.48	2.04 ± 0.29	ND	43.33 ± 0.86

^1^ not detected.

**Table 3:** Concentrations (mg/100 g extract) of compounds in investigated extracts of the *Hypnum cupressiforme* according to the LC-MS analysis.

mg/100 g Extract	E1	E2	E3	E4
Gallic acid	0.62	0.70	0.50	1.21
Protocatechuic acid	3.75	2.89	2.39	3.91
5-*O*-Caffeoylquinic acid	0.14	0.07	0.02	0.04
*p*-Hydroxybenzoic acid	4.56	3.17	5.78	4.62
Caffeic acid	0.65	0.42	0.13	1.10
Quercetin 3-*O*-rutinoside	0.09	0.06	0.01	0.03
*p*-Coumaric acid	2.60	2.33	0.46	4.40
Quercetin 3-*O*-glucoside	0.27	0.21	0.02	0.04
Isorhamnetin 3-*O*-glucoside	0.12	0.06	0.02	0.04
Eriodictyol	0.13	0.11	0.05	0.07
Apigenin	0.51	0.47	0.11	0.11
Naringenin	0.57	0.62	0.12	0.08
Kaempferol	7.35	6.60	0.21	0.47
Acacetin	0.21	0.15	0.09	0.02

## Supplementary Materials

The following are available online, Table S1: Biochemical analysis of *Hypnum cupressiforme* extracts as antioxidant agents. Table S2: Biochemical analysis of *Hypnum cupressiforme* extracts as antidiabetic agents. Table S3: Immunomodulatory potential of *Hypnum cupressiforme* extracts on MRC-5 cells. Table S4: Immunomodulatory potential of *Hypnum cupressiforme* extracts on HCT-116 cells.
