# Supplementary material for: Extracts Characterization and In Vitro Evaluation of Potential Immunomodulatory Activities of the Moss Hypnum cupressiforme Hedw
Source: Molecules. 2020 Jul 23;25(15):3343. doi: 10.3390/molecules25153343 (PMC7436104; doi:10.3390/molecules25153343)
Supplement: Supplementary file 1 [file molecules-25-03343-s001.pdf]

Supplementary material

Extracts Characterization and In Vitro Evaluation of Potential Immunomodulatory Activities of the Moss *Hypnum cupressiforme* Hedw.

Tanja M. Lunić [1], Mariana M. Oalđe [2], Marija R. Mandić [1], Aneta D. Sabovljević [2], Marko S. Sabovljević [2], Uroš M. Gašić [3], Sonja N. Duletić-Laušević [2], Bojan Dj. Božić [1,*,†] and Biljana Dj. Božić Nedeljković [1,*,†]

[1] Institute of Physiology and Biochemistry "Ivan Djaja", Faculty of Biology, University of Belgrade, 11000 Belgrade, Serbia; b3018_2019@stud.bio.bg.ac.rs (T.M.L.); b3022_2019@stud.bio.bg.ac.rs (M.R.M.).

[2] Institute of Botany and Botanical Garden "Jevremovac", Faculty of Biology, University of Belgrade, 11000 Belgrade, Serbia; marianao@bio.bg.ac.rs (M.M.O.); aneta@bio.bg.ac.rs (A.D.S.); marko@bio.bg.ac.rs (M.S.S.); sduletic@bio.bg.ac.rs (S.N.D-L.).

[3] Department of Plant Physiology, Institute for Biological Research "Sinisa Stankovic", National Institute of Republic of Serbia, University of Belgrade, Bulevar despota Stefana 142, 11060 Belgrade, Serbia; uros.gasic@ibiss.bg.ac.rs.

* Correspondence: bbozic@bio.bg.ac.rs (B.D.B); biljana@bio.bg.ac.rs (B.D.B.N); Tel.: +381 11 303 23 56 (B.D.B.N).

Received: date; Accepted: date; Published: date

Supplementary Table 1. Biochemical analysis of *Hypnum cupressiforme* extracts as antioxidant agents.

Samples	Concentration (µg/mL)	Inhibition of DPPH radical (%)	TRP (µmol AAE/g extract)
E1	1000	<5	<5
	500	<5	<5
	100	<5	<5
	50	<5	<5
	10	<5	<5
E2	1000	6.08 ± 0.41	<5
	500	<5	<5
	100	<5	<5
	50	<5	<5
	10	<5	<5
E3	1000	23.80 ± 1.10	13.24 ± 1.87
	500	<5	6.90 ± 0.66
	100	<5	<5
	50	<5	<5
	10	<5	<5
E4	1000	19.22 ± 0.85	10.48 ± 1.09
	500	<5	<5
	100	<5	<5
	50	<5	<5
	10	<5	<5
BHT	1000	84.10 ± 0.15	138.86 ± 1.51
	500	71.70 ± 0.29	123.24 ± 1.03
	100	26.42 ± 0.91	58.05 ± 1.63
	50	12.91 ± 0.65	30.10 ± 1.17
	10	<5	9.90 ± 1.48
BHA	1000	85.16 ± 0.63	118.81 ± 9.57
	500	80.03 ± 0.71	121.38 ± 10.07
	100	29.88 ± 1.27	50.00 ± 2.50
	50	14.53 ± 0.48	26.86 ± 1.98
	10	<5	5.48 ± 0.74
Ascorbic acid	1000	88.11 ± 0.06	129.71 ± 1.51
	500	88.26 ± 0.10	129.05 ± 2.95
	100	87.91 ± 0.06	81.00 ± 2.94
	50	87.65 ± 0.15	58.95 ± 2.25
	10	7.83 ± 0.06	<5

AAE – ascorbic acid equivalents; BHA – butylated hydroxyanisole; BHT – butylated hydroxytoluene; TRP – total reducing power;

Supplementary Table 2. Biochemical analysis of *Hypnum cupressiforme* extracts as antidiabetic agents.

**Samples**	**Concentration (μg/mL)**	**Inhibition of α-amylase (%)**
E1	1000	<5
	500	<5
	100	<5
	50	<5
	10	<5
E2	1000	<5
	500	<5
	100	<5
	50	<5
	10	<5
E3	1000	8.76 ± 0.35
	500	5.68 ± 0.15
	100	<5
	50	<5
	10	<5
E4	1000	<5
	500	<5
	100	<5
	50	<5
	10	<5
Acarbose	1000	82.99 ± 1.18
	500	80.15 ± 1.02
	100	53.65 ± 0.42
	50	47.16 ± 1.25
	10	<5

Supplementary Table 3. Immunomodulatory potential of *Hypnum cupressiforme* extracts on MRC-5 cells.

**Samples**	**Viability (%)**	**ROS production (NBT index)**	**NO production/Nitrite level (μM)**
Control	100.00 ± 2.09	1.00 ± 0.04	54.77 ± 1.99
E1	94.02 ± 1.24*	1.17 ± 0.09	59.04 ± 5.72
E2	86.86 ± 0.46*	0.97 ± 0.03	61.82 ± 2.14*
E3	87.96 ± 1.67*	1.00 ± 0.03	54.58 ± 4.13
E4	105.83 ± 1.79	0.94 ± 0.02	62.91 ± 9.97

The results are expressed as mean ± standard error (*$p<0.05$ different moss extracts *vs.* control).

Supplementary Table 4. Immunomodulatory potential of *Hypnum cupressiforme* extracts on HCT-116 cells.

Samples	Viability (%)	ROS production (NBT index)	NO production/Nitrite level (μM)
Control	100.00 ± 2.16	1.00 ± 0.03	18.35 ± 0.55
E1	89.67 ± 0.68*	1.18 ± 0.05*	20.85 ± 0.30*
E2	86.72 ± 0.35*	1.06 ± 0.03	19.60 ± 0.36
E3	88.59 ± 1.42*	1.22 ± 0.02*	21.21 ± 0.26*
E4	97.59 ± 1.99	1.03 ± 0.05	18.43 ± 0.31

The results are expressed as mean ± standard error (*p<0.05 different moss extracts *vs.* control).
